# Genetic Diversity and Population Demography of the Chinese Crocodile Lizard (*Shinisaurus crocodilurus*) in China

**DOI:** 10.1371/journal.pone.0091570

**Published:** 2014-03-11

**Authors:** Huayuan Huang, Hui Wang, Linmiao Li, Zhengjun Wu, Jinping Chen

**Affiliations:** 1 Key Laboratory of Rare and Endangered Animal Ecology, Guangxi Province, College of Life Science, Guangxi Normal University, Guilin, China; 2 Guangdong Entomological Institute/South China Institute of Endangered Animals, Guangzhou, China; University of Sydney, Australia

## Abstract

The Chinese crocodile lizard *Shinisaurus crocodilurus* is a critically endangered species, listed in Appendix II of CITES. Its populations and habitat in China have undergone significant changes in recent years. Understanding the genetic variability and phylogeography of this species is very important for successful conservation. In this study, samples were taken from 11 wild ponds and two captive populations in China. We sequenced mitochondrial CYTB, partial ND6, and partial tRNA-Glu and genotyped 10 microsatellite loci. Our analyses of these data showed low genetic variability, no strong isolation caused by distance, and a lack of a phylogeographic structure in this species. Based on our results, the basal divergence between two clades of *S. crocodilurus* in China may have been caused by the formation of the Pearl River system. We found a population expansion in one of these clades. Microsatellite analysis indicated the presence of three clusters, separated by significant genetic differences. We found that most individuals in the two captive populations were from the Luokeng (Guangdong) and Guangxi wild source populations, respectively.

## Introduction

The Chinese crocodile lizard, *Shinisaurus crocodilurus*, was first collected in 1928. In 1930, Ahl established a new family (Shinisauridae) to accommodate the monotypic genus and species [1]. The species remains monotypic and is listed in CITES Appendix II [2], [3]. *S. crocodilurus* is currently distributed in the east part of the Guangxi (Kwangsi) Zhuang Autonomous Region, the west and north parts of Guangdong province in southern China, and in mountainous areas of northern Vietnam [4]–[6]. The species is only observed in restricted areas between 200 and 1500 m above sea level along densely vegetated karst streams or ponds.

In recent years, the population size of *S. crocodilurus* has decreased rapidly, and its habitat has been widely destroyed. In the main parts of its distribution, including Jinxiu county, Zhaoping county, and Guiping county, as well as Hexian county in Guangxi (Kwangsi) Zhuang Autonomous Region, *S. crocodilurus* has suffered 70%–80% population declines. The overall number of individuals in China has decreased from 6,000 in 1978 to 950 in 2008 [7], [8]. Currently, less than one-third of the populations in these regions are well protected within nature reserves in China, and illegal hunting still occasionally occurs within these areas. Outside nature reserves, the crocodile lizard’s habitats have been severely damaged. The ideal habitat for the crocodile lizards is broadleaf forest, which maintains water flow in streams year round [2]. However, natural broadleaf forest has been gradually replaced with more profitable trees such as *Illicium verum* and tea shrubs. Such changes in vegetation directly contribute to the decrease in aquatic resources in streams. Small–scale dam construction has changed the distribution of streams and influences the survival of the crocodile lizard. Mining is another source of habitat destruction because it can pollute streams. In these cases, human activities have made the habitat no longer suitable for the crocodile lizard [7].

Although there have been several recent ecological studies of *S. crocodilurus* [9], [10], a description of the genetic structure or relationship of *S. crocodilurus* at different geographic scales has not been conducted. In this study, we analyzed mitochondrial CYTB, partial ND6, and partial tRNA-Glu, as well as microsatellite genetic markers, to determine the evolutionary relationships and structure of *S*. *crocodilurus* populations across the species’ ranges in China. Our primary aims were as follows: (i) to assess the level and partitioning of genetic variation within *S*. *crocodilurus*, (ii) to analyze the demographic history and the events and factors that might have had an influence, (iii) to examine the extent to which habitat fragmentation has affected genetic variation, and (iv) to discuss management strategies for this species.

## Materials and Methods

**Figure 1:**
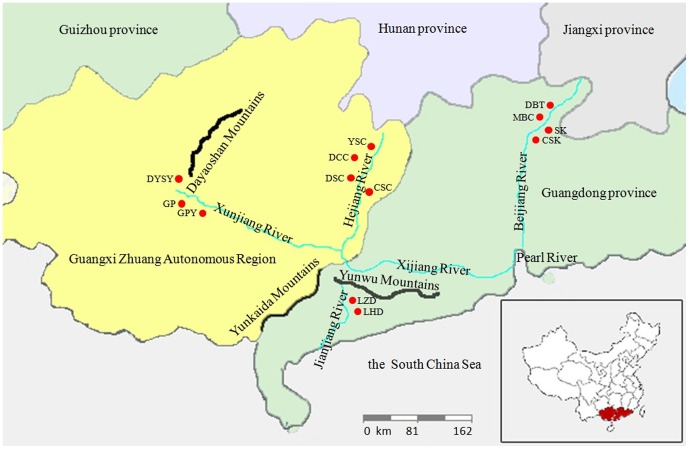
A map of the sample area and site locations in China. Abbreviations are: DYSY, Jinxiu, Guangxi, China (Captive population); DCC, Dacaichong, Hezhou, Guangxi, China; CSC, Chishuichong, Hezhou, Guangxi, China; DSC, Deshengchong, Hezhou, Guangxi, China; YSC, Yusanchong, Hezhou, Guangxi, China; GP, Guiping, Guangxi, China (wild population); GPY, Guiping, Guangxi, China (Captive population); MBC, Miaobei chong, Luokeng, Guangdong, China; CSK, Chishuikeng, Luokeng, Guangdong, China; DBT, Dabeitou, Luokeng, Guangdong, China; SK, Shenkeng, Luokeng, Guangdong, China; LZD, Linzhouding nature reserve, Maoming, Guangdong, China; LHD, Luhuding nature reserve Maoming, Guangdong, China.

Individuals of *S*. *crocodilurus* were captured by hand on clear nights in the field while they were relatively inactive, lying on branches. Saliva samples were collected by buccal swabbing. The cotton swabs used to collect saliva samples were stored in 1.5 ml centrifugal tubes containing 100% ethanol. Saliva samples from 216 individuals were collected from 13 ponds (two captive and 11 wild) throughout the current range of the species in China (Figure 1 and Table 1). After sampling, individuals were immediately returned to where they were captured.

**Table 1:** Characteristics of 13 ponds, and levels of microsatellite DNA and mtDNA variation for *S. crocodilurus.*

pond	Locality	Latitude (N)	Longitude (E)	sample sizes	H_O_	H_E_	F_IS_	*S*	*h*	*π*	Tajima’s *D*	*P*-value
DYSY	Jinxiu, Guangxi, China	24°1′34″	110°14′57″	12	0.57	0.64	0.13	1	0.00	0.0000	0.00	1.000
DCC	Hezhou, Guangxi, China	24°05′56.06″	111°48′13.91″	20	0.40	0.62	0.30	1	0.00	0.0000	0.00	1.000
CSC	Hezhou, Guangxi, China	24°05′44.79″	111°48′32.35″	14	0.32	0.52	0.37	2	0.33	0.0094	−1.52	0.001
DSC	Hezhou, Guangxi, China	24°04′55.57″	111°49′41.88″	10	0.41	0.68	0.42	4	0.73	0.0008	0.00	1.000
YSC	Hezhou, Guangxi, China	24°05′54.79″	111°48′21.08″	24	0.33	0.58	0.40	1	0.00	0.0000	0.00	1.000
GP	Guiping, Guangxi, China	23°33′8″	109°57′39″	9	0.46	0.70	0.35	1	0.00	0.0000	0.00	1.000
GPY	Guiping, Guangxi, China	23°44′19″	110°5’39″	15	0.33	0.68	0.52	1	0.00	0.0000	0.00	1.000
MBC	Luokeng, Guangdong, China	24°34′30″	114°16′50″	14	0.34	0.67	0.50	2	0.33	0.0004	1.03	0.897
CSK	Luokeng, Guangdong, China	24°34′10″	113°16′29″	7	0.45	0.37	0.37	2	0.40	0.0003	−0.97	0.202
DBT	Luokeng, Guangdong, China	24°36′50″	113°21′12″	12	0.37	0.62	0.40	1	0.00	0.0000	0.00	1.00
SK	Luokeng, Guangdong, China	24°35′08″	113°21′14″	7	0.42	0.58	0.29	1	0.00	0.0000	0.00	1.00
LZD	Maoming, Guangdong, China	22°20′	111°29′	40	0.42	0.73	0.41	3	0.80	0.0017	0.96	0.782
LHD	Maoming, Guangdong, China	22°21′57.68″	111°24′46.93″	32	0.51	0.71	0.25	4	0.80	0.0021	0.23	0.621

Expected heterozygosity (H_E_), Observed herterozygosity (Ho) and inbreeding coefficients (F_IS_) are means across all loci. The haplotypes found in each pond are given along with haplotype diversity (h) and nucleotide diversity (π), (S: number of haplotypes observed) and Tajima’s D with its associated *P*-value. Population abbreviations are the same as those in Figure 1.

### Ethical Statement

Sampling was approved by the Forestry Administration of Guangdong province, Dayaoshan Nature Reserve, Daguishan Nature Reserve, Luokeng Nature Reserve, Linzhouding Nature Reserve, and Luhuding Nature Reserve. All lizards were immediately released after the saliva was collected. Buccal swabbing is a noninvasive method. The Committee on the Ethics of Animal Experiments of the Guangxi Normal University and the Guangdong Entomological Institute Administrative Panel on Laboratory Animal Care approved the protocol.

### DNA Extraction, Genotyping, and Sequencing

DNA was extracted using the DNeasy Tissue Kit (Qiagen) according to the manufacturer’s protocol with slight modifications. We amplified 10 microsatellite loci from nuclear DNA using 5′-fluoro-labeled forward primers (MedProbe) [11]. The PCR amplifications were performed in 15-μl reactions containing approximately 1 μl of template DNA, 1 μl of each primer, 4.5 μl of H_2_O, and 7.5 μl of Premix Taq DNA polymerase (Takara). The amplification conditions were as follows: 95°C for 5 min, 35 cycles at 94°C for 35 s, T_a_ (55°C–61°C) for 35 s (Table S1), 72°C for 40 s, and a final extension at 72°C for 10 min (Supplementary Table 1). Allele sizing was performed at the Beijing Genomics Institute (BGI) using automated fluorescent scanning detection with an ABI 377XL DNA sequencer (Applied Biosystems) with ROX500 as an internal lane size standard and Genescan and Genotyper (Applied Biosystems) software.

A subset of 13 ponds selected to represent each region was used for the mitochondrial DNA analysis, and five individuals from each pond were sampled. A 1364 bp region of the mitochondrial DNA (CYTB, partial ND6, and partial tRNA-Glu) gene was amplified using primer 1 (5′GCAATTGAATAAGCAAAAACCAC3′) and primer 2 (5′TAGTTTATTAAAAATGCTAGTTTTGGG3′). Amplification was performed in a total volume of 30 μl containing approximately 2 μl of template DNA, 1 μl of each primer, 11 μl of H_2_O, and 15 μl of Premix Taq DNA polymerase (Takara). Initial denaturation was performed at 95°C for 5 min, followed by 35 cycles for 45 s at 94°C, 1 min at 56°C, and 1 min at 72°C, with a final elongation step for 10 min at 72°C. The PCR product was run on a 1.5% agarose gel for 30 min at 100 V. The PCR products were purified using a PCR purification kit (Shanghai Bio-Tec, Ltd.), sequenced using the ABI PRISM BigDye Terminator Ready Reaction kit (Applied Biosystems), and run on an ABI 377 genetic analyzer according to the manufacturer’s protocol.

### Analysis of Microsatellite Data

Microsatellite variation within each pond was measured using allele frequency data, from which the average allelic richness, inbreeding coefficient (Fis), and gene diversity were calculated using the method of Arlequin [12]. Genetic divergence between the ponds was estimated by calculating pairwise *p* values, which represents an unbiased estimate of Fst [13]. Estimates of genetic diversity within populations, departures from the Hardy–Weinberg equilibrium, and pairwise h-values were all analyzed using Fstat [14]. To assess the spatial genetic structure of the populations, we analyzed the correlation between genetic divergence and geographic distance using the Isolation by Distance v1.52 program [15].

Population structure was also analyzed with STRUCTURE 2.3.3 [16]. The Bayesian clustering method was used to detect structure in the whole dataset and to assign individuals to inferred clusters. Five independent runs of *K* = 1–10 were performed using 1,000,000 Markov Chain Monte Carlo steps, with a burn-in period of 100,000. We used no prior information and assumed correlated allele frequencies and admixture. The log likelihood was used to select the most likely value for K. In addition, we estimated the Δ*K* statistic, which measures the second-order rate of change in the log likelihood of the data between successive values of *K* [17]. This method can be used to estimate the appropriate number of clusters for simulated data sets under a number of gene-exchange models. Because one cannot evaluate Δ*K* for *K* = 1, we investigated K = 2 to K = 9.

The significance of the hierarchical partitioning of genetic structure among the geographic groups was examined using an analysis of molecular variance (AMOVA). This analysis was done in Arlequin [12].

### Analysis of Mitochondrial Sequence Data

The DNA sequence data were edited using Sequencer v4.10.1 (Gene Codes Corporation, Ann Arbor, MI, USA, http://www.genecodes.com). The sequences were then aligned using Clustal X [18], and all alignments were visually inspected. Departures from neutrality for each population were assessed using Tajima’s statistic as implemented in Arlequin v3.1 [12], [19]. Haplotypes were estimated using DnaSP [20], and the nucleotide diversity (*π*), haplotype diversity (*h*), and number of polymorphic sites (*S*) within each pond was also calculated in DnaSP [20]. We used NETWORK 4.6 [21] to infer maximum-parsimony networks with 95% connection limits; the concatenated mtDNA sequences were used to reconstruct the median-joining network, and the geographic distribution of the haplotypes was overlaid on the networks.

Bayesian phylogenetic analyses of the mitochondrial sequences were conducted using MrBayes v3.1.2 [22]. *Lepidophyma flavimaculatum* (GenBank: AB122908.1), *Uromastyx benti* (NCBI Reference Sequence: NC 014182.1), *Takydromus tachydromoides* (NCBI Reference Sequence: NC_008773.1), *Gekko gecko* (NCBI Reference Sequence: NC_007627.1), and *Phrynocephalus mystaceus* (NCBI Reference Sequence: NC_021131.1) were used as outgroups. The best-fitting substitution model was determined using MODELTEST 3.06 [23], Monte Carlo Markov chains were run for 20,000,000 generations, sampling every 100 generations, and the initial 10% of trees were discarded as burn-in.

Genetic structure among geographic regions was assessed using the AMOVA method in Arlequin. The AMOVA calculations were based on haplotype frequencies and pairwise differences between the haplotypes. The population history was inferred from a comparison of π and Tajima’s D test of neutrality using Arlequin and DnaSP v3.0 [20]. Population expansion was detected by DnaSP v3.0 [20].

## Results

### Microsatellite Data

Micosatellite genotyping results are given in Table S2. As shown in Table 2, the estimates of microsatellite genetic variation differed among the populations, with H_E_ values ranging from 0.300 to 0.735. The average inbreeding coefficient (Fis) was 0.3, and ranged from 0.134 to 0.504. All Fis values were greater than zero.

**Table 2:** Hierarchical analysis of molecular variance (AMOVA) apportioned among geographical regions, ponds, and individuals.

Source of variation	Percentage of variation	Fixation indices	*P*-value
Microsatellites			
Among regions	4.53	Fct:0.0452**	0.0078
Among ponds within regions	12.85	Fsc:0.1345***	0.0000
Among individuals within ponds	30.28	Fis:0.3664***	0.0000
Within individuals	52.35	Fit:0.4765***	0.0000
MtDNA (Haplotype frequencies)			
Among regions	80.90	Fct:0.7888**	0.0019
Among ponds within regions	−0.35	Fsc:0.0018	0.5372
within ponds	19.45	Fst:0.7931***	0.0000

*p<0.05; **p<0.01; ***p<0.001.

**Figure 2:**
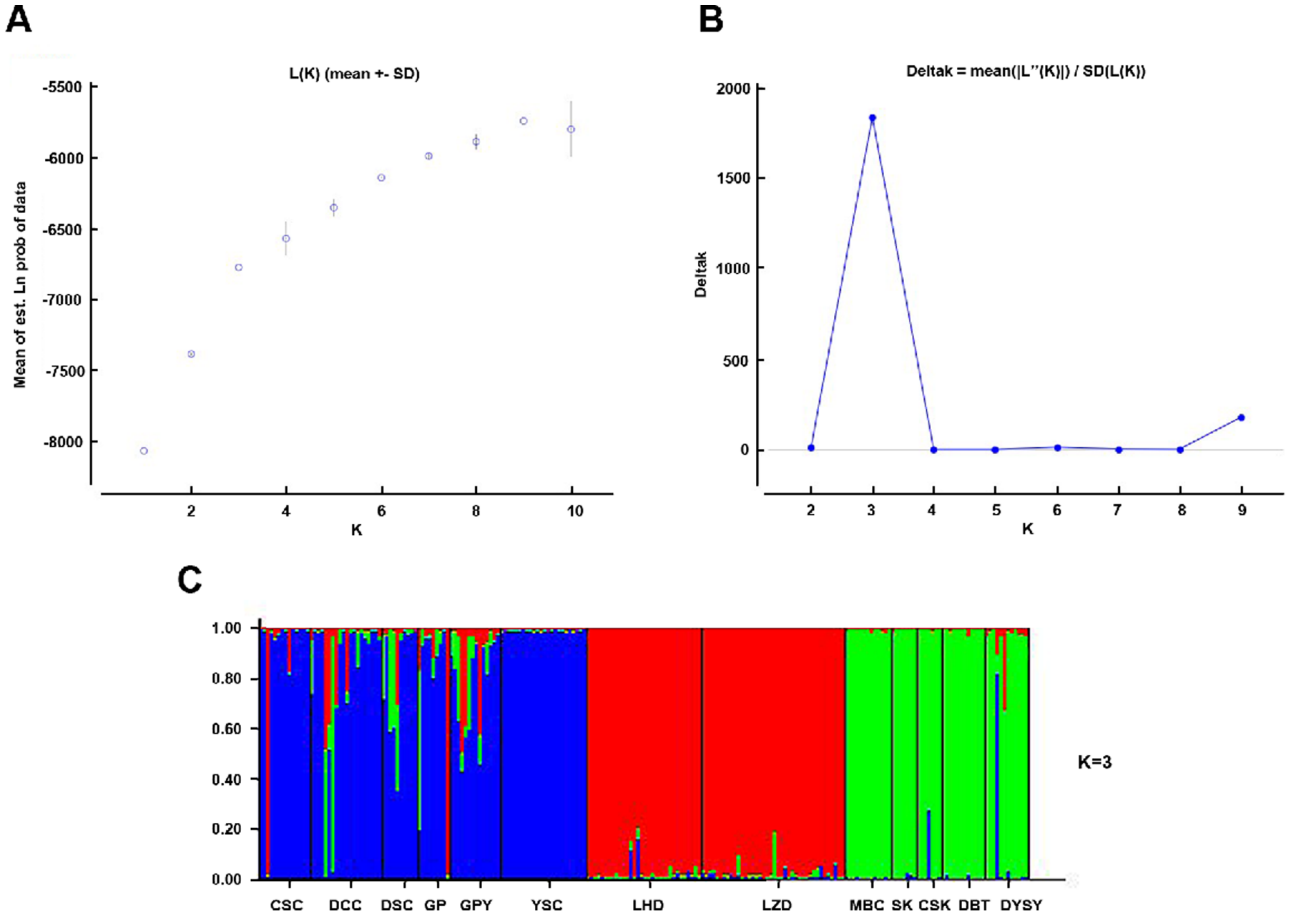
Population structure was determined from microsatellite data using the STRUCTURE software when *K* = 3. A: Mean L(*K*)(±SD) five runs for each value of *K* from 1 to 10.; B: *ΔK* was calculated as *ΔK* = *m*(|L’’(*K*)|)/*s*[L*(K*)], and *K* = 3 had the highest *ΔK* versus *K* peak height; C: A bar plot of the *K* = 3 estimates. Each individual is represented by a single vertical line broken into *K* colored segments, with length proportional to each of the *K* inferred clusters. CSC, DCC, DSC, GP, GPY, and YSC were clustered in the Guangxi population (GX); MBC, CSK, DBT, SK, and DYSY were clustered in the LK population; LZD and LHD were clustered in the MM population. Population abbreviations are the same as those in Figure 1.

We used a clustering algorithm in STRUCTURE to infer the relationships between populations. The most likely population structure was observed for *K* = 3 and separates the three hydrographic systems (Figure 2): GX (the Guangxi population), LK (Luokeng, Guangdong population), and (Maoming, Guangdong population).

Hierarchical structuring was also observed using an AMOVA. Significant microsatellite variance was observed between regions (4.53%, *p* = 0.007), between populations within each region (12.85%, *p* = 0.000), and within populations (30.28%, *p* = 0.000), indicating that greater genetic variance was partitioned within individuals (52.35%, *p* = 0.000) than between populations (30.28%) (Table 2).

### Mitochondrial Sequence Data

**Figure 3:**
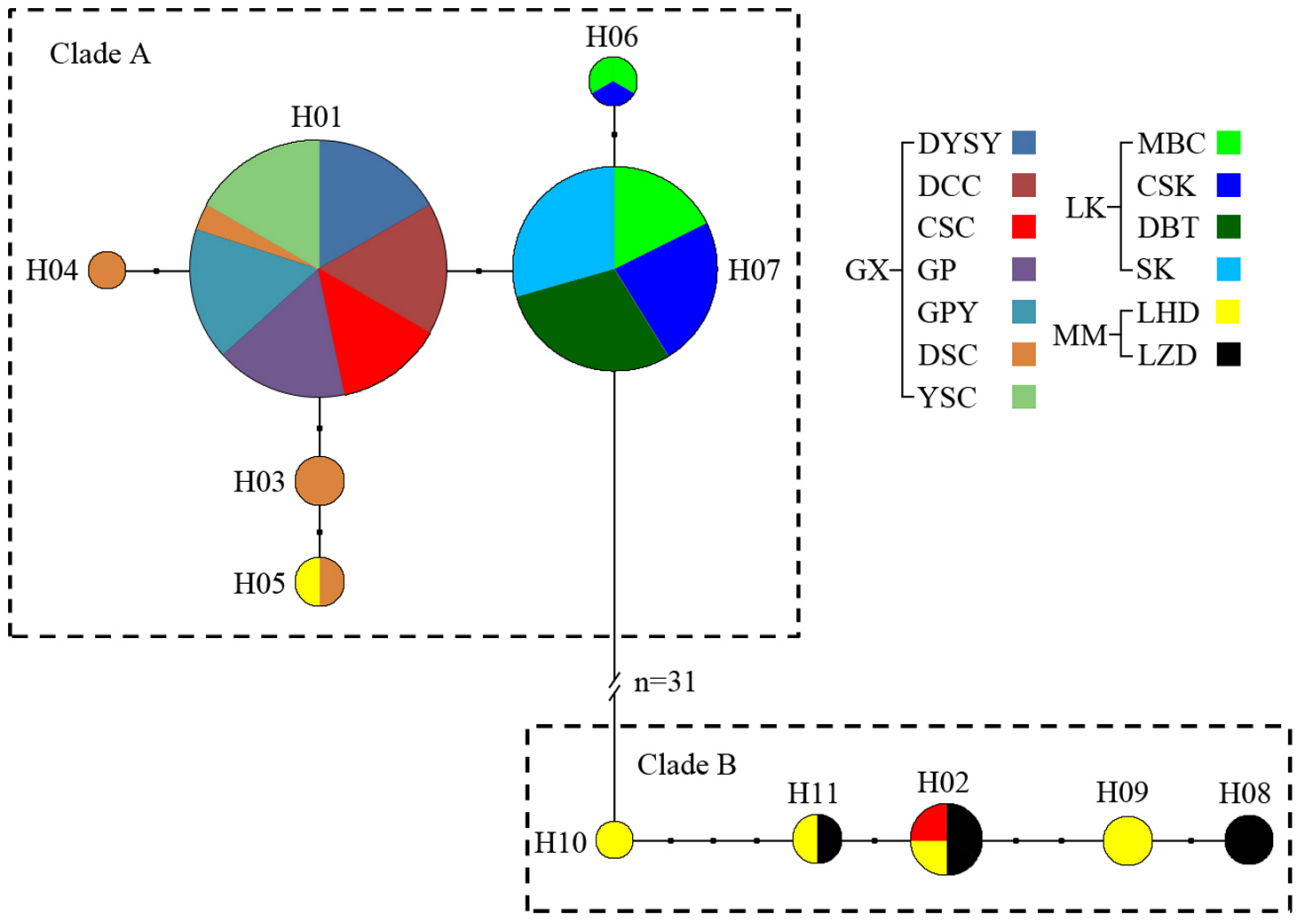
Five individuals were sequenced from each pond, with the median-joining network showing phylogenetic relationships among populations. Each haplotype is shown as a circle, the size of which indicates the number of individuals with that haplotype (haplotypes within each population are provided in Table S3). Mutational steps connecting haplotypes are represented by a small black circle between haplotypes. Different colors represent the different populations (abbreviations are the same as those in Figure 1).

Of the 65 mitochondrial DNA samples from *S*. *crocodilurus* individuals, 11 unique haplotypes were observed (Figure 3) and their GenBank accession numbers are KF928266-KF928276. Diversity indices *h* (haplotype diversity) and *π* (nucleotide diversity) are summarized in Table 1. On average, *h* was 0.710 and *π* was 0.007.

Hierarchical structuring was also determined using an AMOVA. Significant variance was observed among regions (80.90%, *p* = 0.001) and within ponds (19.45%, *p* = 0.000), but no significant variance was found among ponds within each region (−0.35%, *p* = 0.537). This indicates that greater genetic variance exists among regions than among ponds within each region (Table 2). In the analysis of isolation by distance, the genetic and geographic distances did not show any significant correlations in any of the geographic regions (*r* = −0.2958, *p* = 1.00).

### Phylogenetic Analysis

The GTR+I+G model was identified as the best-fitting substitution model. Phylogenetic analyses showed there were two main haplogroups, clades A and B (Figure 3). Clade B contained haplotypes H02, found in LHD (Luhuding Nature Reserve), DSC (Deshengchong, Hezhou, Guangxi, China), and LZD (Linzhouding Nature Reserve); H08, found in LZD; H09, found in LHD; H10, found in LHD and DSC; and H11, found in LZD and LHD. The other six haplotypes were placed in clade A (Figure 3). It should be noted that haplotypes in clade B occurred in the MM and GX populations, and haplotypes in clade A were also found in the LK and GX populations. The mismatch distribution strongly indicates a past population expansion within clade A. In contrast, the population growth parameter indicates a stable demographic history in clade B. The median-joining network for 11 haplotypes produced a relatively simple pattern (Figure 3), without obvious star-like topological structures in the network maps and without central haplotypes.

## Discussion

### Genetic Diversity

The Chinese crocodile lizard (*S*. *crocodilurus*) shows low genetic diversity, with a mitochondrial DNA nucleotide diversity (***π***) level of 0.007 and an average expected heterozygosity (H_E_) of 0.625 for the microsatellite loci. The nucleotide diversity of *S*. *crocodilurus* is lower than that of the ornate dragon lizard, *Ctenophorus ornatus* (a habitat-specializing lizard, *π* = 0.14±0.05) [24], and *Gnypetoscincus queenslandiae* (suffering human-induced habitat fragmentation, *π* = 0.408±0.05) [25]. The microsatellite heterozygosity levels (H_E_ = 0.583) are lower than those in populations of the ornate dragon lizard (H_E_ = 0.77) [24], *Oedura reticulata* (H_E_ = 0.79), and *Gehyra variegata* (H_E_ = 0.88, persistence in habitat remnant) [26] but were slightly higher than those of the Swedish sand lizard, *Lacerta agilis* (facing fragmentation threat, H_E_ = 0.45) [27], and *Mintonius gloydi* (which crosses a fragmented landscape, H_E_ = 0.53) [28].

Our study shows that *S. crocodilurus* has low genetic diversity in China and highlights the need for conservation efforts. Previous studies based on morphological characteristics implied a high genetic diversity for *S. crocodilurus* [29], which may reflect the use of smaller sample sizes and less powerful markers than those used in the present study.

### Genetic Structure

Mitochondrial DNA haplotypes in *S. crocodilurus* displayed local homogeneity and strong population structure; with all the LK haplotypes and the majority of the GX haplotypes placed in clade A, and the majority haplotypes of the MM in clade B (Figure 3). Such distributions for mitochondrial DNA haplotypes have also been observed in the ornate dragon lizard [24]. Based on the results of our NETWORK analyses and previous field surveys, we hypothesize that the basal divergence between clades A and B may have been caused by the development of the Pearl River system, as well as the formation of the Jianjiang River system [29]. Populations from clade A are found along the Pearl River system, whereas populations from clade B are located south of the Yunwu Mountains, an area included in the Jianjiang River system [29]. The geological barrier presented by the Yunwu Mountains may have led to the differentiation of these two clades [30].

The hierarchical STRUCTURE results from the microsatellite markers divide the Guangdong population into two clusters (LK and MM), as shown in Figure 3. An AMOVA for both mitochondrial and nuclear DNA showed significant genetic differentiation between the regions and among the ponds within each region (Table 2). These results were also supported by the clustering analysis.

Genetic diversity is maintained by large population sizes and/or gene flow within populations [31]. The significant genetic divergence and genetic structure of *S. crocodilurus* suggest that there has been gene flow only over short distances.

### Phylogenetic and Geological History

The distribution of *S. crocodilurus* is discontinuous [6], and the origin and phylogeography of the species remain unclear [32]. We were unable to observe any evident geographic origin for *S. crocodilurus*, which is consistent with previously published studies. Gong et al. reported that the distribution of *S. crocodilurus* could be connected by the Pearl River system, with the exception of the MM [29], [32]. The GX population was distributed with the stream drainage (Hejiang River and Xunjiang River) into the Pearl River system, and the LK populations were distributed with the drainage into the Beijiang River of the Pearl River system (Figure 1). However, the MM population originated from the Linzhouding and Luhuding Nature Reserves, which are located in the Yunwu Mountains (Figure 1). In addition, the *S. crocodilurus* population distribution in China may have been divided into two clades because the Yunwu Mountains are the watershed of the Pearl River system and Jianjiang River drainage.

During the Cenozoic era, Dayaoshan Mountain was located in the central region of the Guangxi Arcuate Mountains, which was an important pathway of animal migration in Guangxi province. Dayaoshan Mountain is at the intersection of the north-south species transition, and an ice sheet was not present during the Quaternary glacial period. Therefore, this intersection was an ancient species refuge with rich species diversity [32]. Our study reveals that the most widespread and frequent haplotypes in clade A (H01 and H07) are distributed in GX and LK (Figure 3), suggesting that this might have been the location of an initial population expansion.

Restoring wildlife populations in the field by captive breeding is helpful for many endangered animals. According to our study, the individuals in the two captive populations, DYSY (Jinxiu, Guangxi, China) and GPY (Guiping, Guangxi, China), were derived from the LK and GX wild populations, respectively. The average expected heterozygosity of the two captive groups (H_E_ = 0.66) was similar to that of the wild groups (H_E_ = 0.61), and the average Fis values of the captive groups (0.33) were also similar to those of the wild groups (0.37) (Table 2). Captive populations did not show changes in the level of genetic diversity, and this study also supports the need to conserve *S. crocodilurus* captive populations to restore this species.

This study is the first to assess the genetic diversity in *S. crocodilurus* on a large scale and includes 13 ponds throughout the studied range. The 11 haplotypes had low haplotype diversity (0.710), and the nucleotide diversity was also low (0.007). According to our study, all ponds had Fis values greater than zero, demonstrating that *S. crocodilurus* is extensively inbred. Urgent actions should be taken to protect this species.

### Conclusions

Management units are identified by significant differences in the allele frequency distributions and significant divergence in mitochondrial or nuclear loci [33]. Considering these criteria, populations with genotypes that are closely related to but not shared with other populations are described as management units. Our analyses have shown that *S. crocodilurus* comprises three main genetic groups; GX, LK, and MM, which correspond to distinct geographic populations of Guangxi, China; Luokeng, Guangdong, China; and Maoming, Guangdong, China, respectively. On this basis, we propose the following three management units: [CSC, DCC, DSC, YSC], [MBC, CSK, DBT, SK], and [LZD, LHD] (Figure 1). Protection of all three management units is required because of their low genetic diversity. DSC is the most important population, with three unique haplotypes that are different from all other populations. We found that captive breeding populations did not have a lower genetic diversity than wild populations because of frequent introductions of wild individuals into the farms.

## Supporting Information

Table S1 **Characterization of 10 microsatellite loci in** ***Shinisaurus crocodilurus.***
(DOC)

Table S2 **Microsatellite genotyping raw data, abbreviations are the same as those in** **Figure 1** **.** Locuses are the same as those in Table S1.
(XLS)

Table S3 **Summary of mtDNA CYTB, partial ND6, and partial tRNA-Glu region haplotypes distribution, abbreviations are the same as those in** **Figure 1** **.**
(DOC)
