# Supplementary material for: Genetic Diversity and Population Demography of the Chinese Crocodile Lizard (Shinisaurus crocodilurus) in China
Source: PLoS One. 2014 Mar 11;9(3):e91570. doi: 10.1371/journal.pone.0091570 (PMC3950216; doi:10.1371/journal.pone.0091570)
Supplement: Table S1 — Characterization of 10 microsatellite loci in Shinisaurus crocodilurus. (DOC) [file pone.0091570.s001.doc]

Supplementary Table1 ：Characterization of 10 microsatellite loci in *Shinisaurus crocodilurus*

| **Locus/**  **Accession No.** | **Primer sequence (5’-3’)** | **Core sequences** | ***T*_a_ (ºC)** | **No. of alleles** | **Size range of alleles (bp)** | **GenBank**  **accession No.** |
| --- | --- | --- | --- | --- | --- | --- |
| EX01 | F: CATCAGCCTGGAAAGACTCA | (GT)_18_ | 60 | 15 | 231-259 | JQ411760 |
|  | R: GACAGTTATTCGTTAGGTGGAA |  |  |  |  |
| EX03 | F: TCAACGAACCATTTCAGC | (GT)_17_N_n_(CA)_5_ | 58 | 14 | 271-299 | JQ411758 |
|  | R: CATGTCATGCGAACAAGC |  |  |  |  |
| EX04 | F: CCCAGGTAGGCTTGTATG | (GT)_17_ | 59 | 11 | 238-278 | JQ411757 |
|  | R: TGACCACCGACCAGTTAT |  |  |  |  |
| EX06 | F: GCATATTAAGAACTGGAGCCT | (GT)_14_ | 55 | 16 | 194-248 | JQ411755 |
|  | R: GTGCAGCCCACATGATTG |  |  |  |  |
| EX07 | F: ACAAGCTGGAACTCAAGGG | (GT)_24_ | 59 | 16 | 188-232 | JQ411754 |
|  | R: TGACATACACGAAATAACGAAT |  |  |  |  |
| EX08 | F: TGAGTGCGTGTATGTGTAT | (TG)_5_N_n_(TG)_24_ | 59 | 19 | 190-254 | JQ411753 |
|  | R: ATCCGTCCCGATGAAATG |  |  |  |  |
| EX09 | F: TGGAAGGCAAAGTGGTGA | (AC)_23_ | 59 | 16 | 169-199 | JQ411752 |
|  | R: GAGGGATGGAAATGAGTGAG |  |  |  |  |
| EX10 | F: CCTGTATCCTCCCCTCCT | (CA)_27_ | 59 | 16 | 250-282 | JQ411751 |
|  | R: TGCTGCACTGTGCCTATT |  |  |  |  |
| EX11 | F: AGCAATGAGCAGGACTGAA | (CA)_6_N_n_(AC)_20_ | 61 | 11 | 156-182 | JQ411750 |
|  | R: ACATGCTGAGATGGAGGG |  |  |  |  |
| EX12 | F: GGCACTTCTTCCTCTTAC | (TG)_20_ | 58 | 13 | 185-209 | JQ411749 |
|  | R: TCTCACTGGAGCATTTTG |  |  |  |  |

F: former primer; R: reverse primer; *T*_a_: annealing temperature;
