# Supplementary material for: Genetic Diversity and Population Demography of the Chinese Crocodile Lizard (Shinisaurus crocodilurus) in China
Source: PLoS One. 2014 Mar 11;9(3):e91570. doi: 10.1371/journal.pone.0091570 (PMC3950216; doi:10.1371/journal.pone.0091570)
Supplement: Table S3 — Summary of mtDNA CYTB, partial ND6, and partial tRNA-Glu region haplotypes distribution, abbreviations are the same as those in Figure 1 . (DOC) [file pone.0091570.s003.doc]

Supplementary Table 2 ：Summary of mtDNA CYTB region haplotype distribution

|  | DYS-Y | DCC | CSC | DSC | YSC | ZP | GP | GP-Y | MBC | CSK | YHJ | DBT | SK | JGC | STT | AL | LZD | LUD | TOT |
| --- | --- | --- | --- | --- | --- | --- | --- | --- | --- | --- | --- | --- | --- | --- | --- | --- | --- | --- | --- |
| Hap01 | 5 | 5 | 5 | 5 | 5 |  | 5 | 5 |  |  |  |  |  |  |  |  |  |  | 35 |
| Hap02 |  |  | 1 |  |  |  |  |  |  |  |  |  |  |  |  |  | 2 | 1 | 4 |
| Hap03 |  |  |  |  |  | 2 |  |  |  |  |  |  |  |  |  |  |  |  | 2 |
| Hap04 |  |  |  |  |  | 1 |  |  |  |  |  |  |  |  |  |  |  |  | 1 |
| Hap05 |  |  |  |  |  | 2 |  |  |  |  |  |  |  |  |  |  |  |  | 2 |
| Hap06 |  |  |  |  |  |  |  |  | 2 | 1 |  |  |  |  |  |  |  |  | 2 |
| Hap07 |  |  |  |  |  |  |  |  | 4 | 6 | 3 | 4 | 5 | 2 | 1 | 2 |  |  | 27 |
| Hap08 |  |  |  |  |  |  |  |  |  |  |  |  |  |  |  |  | 2 |  | 2 |
| Hap09 |  |  |  |  |  |  |  |  |  |  |  |  |  |  |  |  |  | 3 | 3 |
| Hap10 |  |  |  |  |  |  |  |  |  |  |  |  |  |  |  |  |  | 1 | 1 |
| Hap11 |  |  |  |  |  |  |  |  |  |  |  |  |  |  |  |  | 1 | 1 | 2 |
| TOT | 5 | 5 | 5 | 5 | 5 | 5 | 5 | 5 | 6 | 7 | 3 | 4 | 5 | 2 | 1 | 2 | 5 | 6 | 82 |
